# Mapping the concept of vulnerability related to health care disparities: a scoping review

**DOI:** 10.1186/1472-6963-13-94

**Published:** 2013-03-12

**Authors:** Cristina Grabovschi, Christine Loignon, Martin Fortin

**Affiliations:** 1 Centre de Recherche Hôpital Charles LeMoyne, Unité de Médecine de Famille HCLM, 150 Place Charles Lemoyne, Bureau 200, Longueuil, QC, J4K 0A8, Canada; 2 Unité de Médecine de Famille, 305 St-Vallier, Chicoutimi, QC, G7H 5H6, Canada

**Keywords:** Vulnerability, Health care disparities, Inverse care law, Scoping review

## Abstract

**Background:**

The aim of this paper is to share the results of a scoping review that examined the relationship between health care disparities and the multiplicity of vulnerability factors that are often clustered together.

**Methods:**

The conceptual framework used was an innovative dynamic model that we developed to analyze the co-existence of multiple vulnerability factors (multi-vulnerability) related to the phenomenon of the ‘Inverse Care Law’. A total of 759 candidate references were identified through a literature search, of which 23 publications were deemed relevant to our scoping review.

**Results:**

The review confirmed our hypothesis of a direct correlation between co-existing vulnerability factors and health care disparities. Several gaps in the literature were identified, such as a lack of research on vulnerable populations’ perception of their own vulnerability and on multimorbidity and immigrant status as aspects of vulnerability.

**Conclusions:**

Future research addressing the revealed gaps would help foster primary care interventions that are responsive to the needs of vulnerable people and, eventually, contribute to the reduction of health care disparities in society.

## Background

Health care disparities are well studied and documented problems that generally refer to differences in the quality of health care - in terms of access, treatment options, prevention and health outcomes - across groups that reflect social inequalities [1-6]. Segments of the population at risk of poor health and health care disparities are usually considered as being vulnerable [7,8]. The concept of vulnerability has become increasingly popular in the scientific literature over the last decades. Rooted in a large array of disciplines such as economics, sociology, anthropology, environmental science and health, these papers refer to various definitions and measures of vulnerability [9]. In regard to the health literature specifically, most of the papers use the concept of vulnerability to indicate the potential risk of developing certain diseases or suffering from environmental hazards. Nevertheless, there is also a fairly large number of publications devoted exclusively to the study of vulnerable populations. These publications have generally introduced and debated conceptual models as frameworks for studying the origin and the consequences of vulnerability on poor health [8,10]. Although these papers sometimes suggest that people who are at risk of poor health are also more likely to face health care disparities, they do not usually focus on studying those disparities. They also do not explicitly examine the link between health care disparities and the co-existence of multiple aspects of vulnerability. Thus, given the broad scope and rapidly growing scientific evidence, we argue that there is a need for a critical review of the literature aiming to grasp the strengths and weaknesses of the current state of knowledge.

The aim of the present review was to examine the concept of vulnerability in connection with the health care disparities faced by distinct subpopulations generally viewed as vulnerable. Furthermore, our main purpose was to determine what is known, from the existing literature, about the relationship between health care disparities and the multiplicity of risk factors that are often clustered together and acting synergistically in the same individual.

To reach these objectives, we used the scoping review as an investigative method. The scoping review originated in the work of Arksey and O’Malley from the Centre for Reviews and Dissemination at the University of York [11] and is becoming an increasingly popular method of reviewing health research evidence. Scoping reviews are used to methodically describe the size and nature of the evidence base for a particular topic area, which can in turn be used to identify gaps in the literature and make recommendations for future primary research [11-15]. Scoping reviews generally differ from systematic reviews: (1) by addressing broader and more heterogeneous questions; (2) by including studies of many different methodological designs; and (3) by not necessarily assessing the quality of the included studies [11-13,15,16]. Thus, the advantages of a scoping review lie in its flexibility and creativity, since it aims to give meaning to the “what” and “why” as opposed to the “who”, “where” and “how” that are specific to the systematic review [14]. The scoping review was our method of choice mainly because, given the exploratory nature of our research question, it was not possible for us to exclude heterogeneous evidence (e.g. qualitative, non-research) whose quality could not be easily appraised by the traditional methods used for systematic reviews.

## Methods

This scoping review is premised on the phenomenon termed the Inverse Care Law (ICL) [17,18], which states that the people with the greatest health care needs receive the least health care services. There is a growing body of research evidence indicating that the socioeconomically deprived are less likely to have a regular physician and more likely to report difficulties obtaining needed primary, secondary and/or preventive care [19,20]. Although this unfortunate paradox of the availability of good medical care, which tends to vary inversely with the need for it, has already been well studied and documented [19], there is still little research on how the ICL operates. Moreover, few studies have examined the combined influence of multiple risk factors on obtaining needed health care services [21].

**Figure 1:**
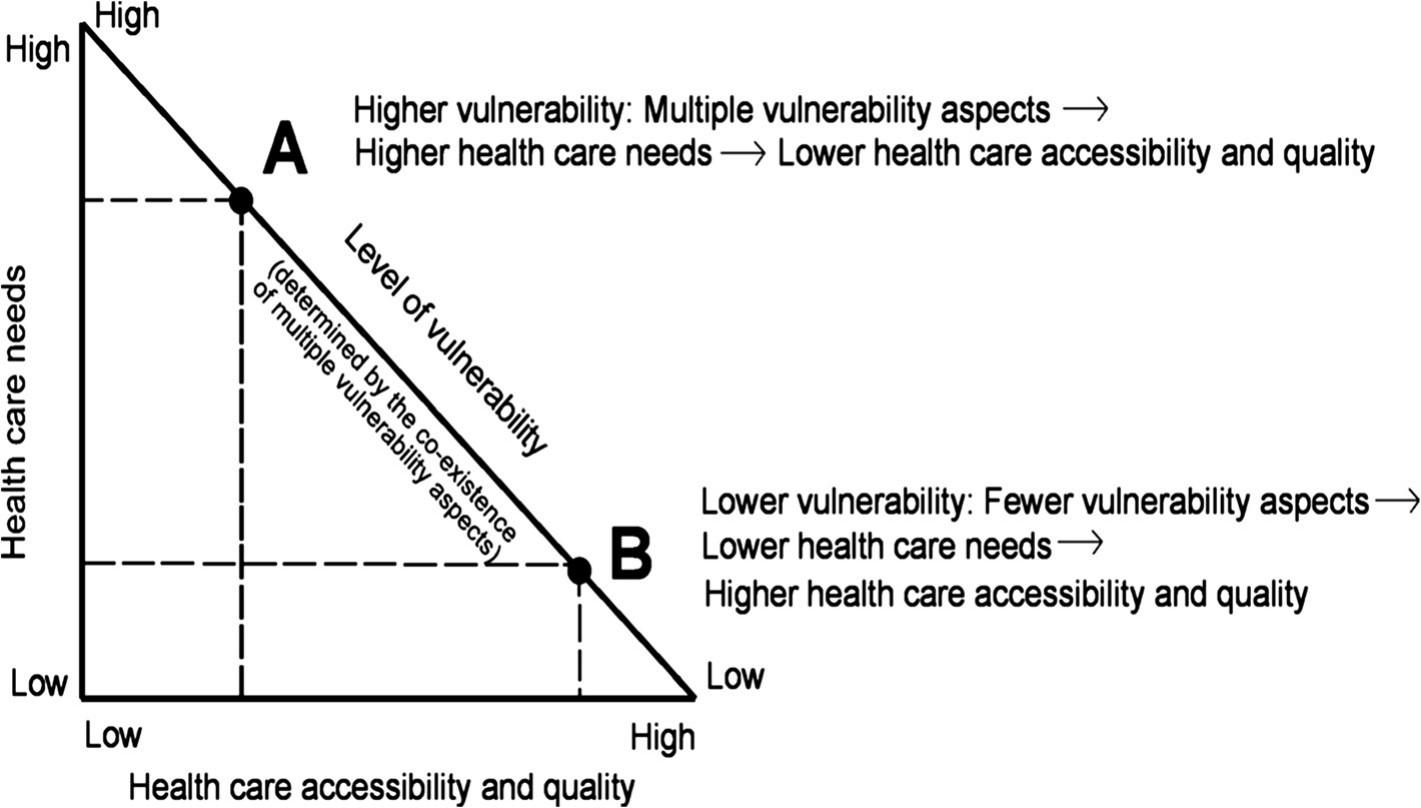
The dynamic multi-vulnerability model of health care disparities.

Based on the evidence-based phenomenon of the ICL, we propose a conceptual model of multi-vulnerability that illustrates the dynamic relationship between health care services received, needs, and level of vulnerability (Figure 1).

The model is a right-angled triangle whose base (horizontal cathetus) represents the continuum of health care accessibility and quality, which can vary from low to high. The vertical cathetus represents the continuum of health care needs, which also vary from low to high depending on the number of vulnerability factors that co-exist in the same individual at a given moment. The triangle’s hypotenuse corresponds to the level of vulnerability that tends to vary directly with health care needs and inversely with accessibility and quality of care. For instance, an individual (A) would experience high vulnerability because of the co-existence of multiple vulnerability aspects, which would result in higher health care needs and be associated, according to the ICL, with lower health care accessibility and quality. Conversely, an individual (B) would experience low vulnerability if he/she presented fewer vulnerability aspects and, therefore, would have low health care needs associated with higher accessibility and quality of health care.

Even though the ICL was originally used to explain inequalities in health care faced by the socioeconomically deprived, we hypothesize that other vulnerability aspects would be subject to the same phenomenon. Moreover, an increase in co-existing vulnerability factors would also be correlated with an increase in health care disparities.

We operationalize vulnerability as an increased susceptibility to health and health care disparities due to a combination of individual and environmental factors. The individual characteristics can be either inborn (e.g., gender, race, genetic predispositions to disease) or acquired (e.g. trauma, diseases, lifestyle), while the environmental aspects refer either to the immediate physical environment (e.g., temperature, pollution, housing, community and neighborhood characteristics) or to the broader socioeconomic environment (e.g., social networks, historical, political, and cultural context) [7,8,22,23]. Our conceptualization of vulnerability is also inspired by the community social resources model by Flaskerud and Winslow, which suggests that vulnerability stems from a lack of socioeconomic and environmental resources. The socioeconomic resources refer to human capital (i.e. income, jobs, education and housing), social connectedness and social status. The environmental resources are operationalized as access and quality of health care and concern the characteristics of the community and the availability of health care professionals and social service providers [24].

This study’s method was based on the scoping review framework outlined by Arskey and O’Malley [11] and encompasses five stages: identifying the search question, identifying the relevant studies, selecting the studies, charting the data, and summarizing and reporting the results.

### Identifying the research question

The central question of this scoping review was: How is the concept of vulnerability used in the existing literature, and what is known about the relationship between health care disparities and the co-existence of multiple aspects of vulnerability in the same population?

### Identifying the relevant studies

The evidence was searched by way of electronic databases (MEDLINE-Ovid, CINAHL, EMBASE and PsycINFO), reference lists, and by hand-searching key journals. The key words used were “vulnerability”, “vulnerable”, “health care”, “healthcare” and “disparities”. Relevant publications were defined as any theoretical or empirical peer-reviewed paper, published since 1990 in English or French, and satisfying the following inclusion criteria: (1) Papers that refer to the concept of vulnerability; (2) Papers that refer to health care disparities; (3) Papers that refer to the co-existence of two or more aspects of vulnerability.

### Selecting the studies

**Figure 2:**
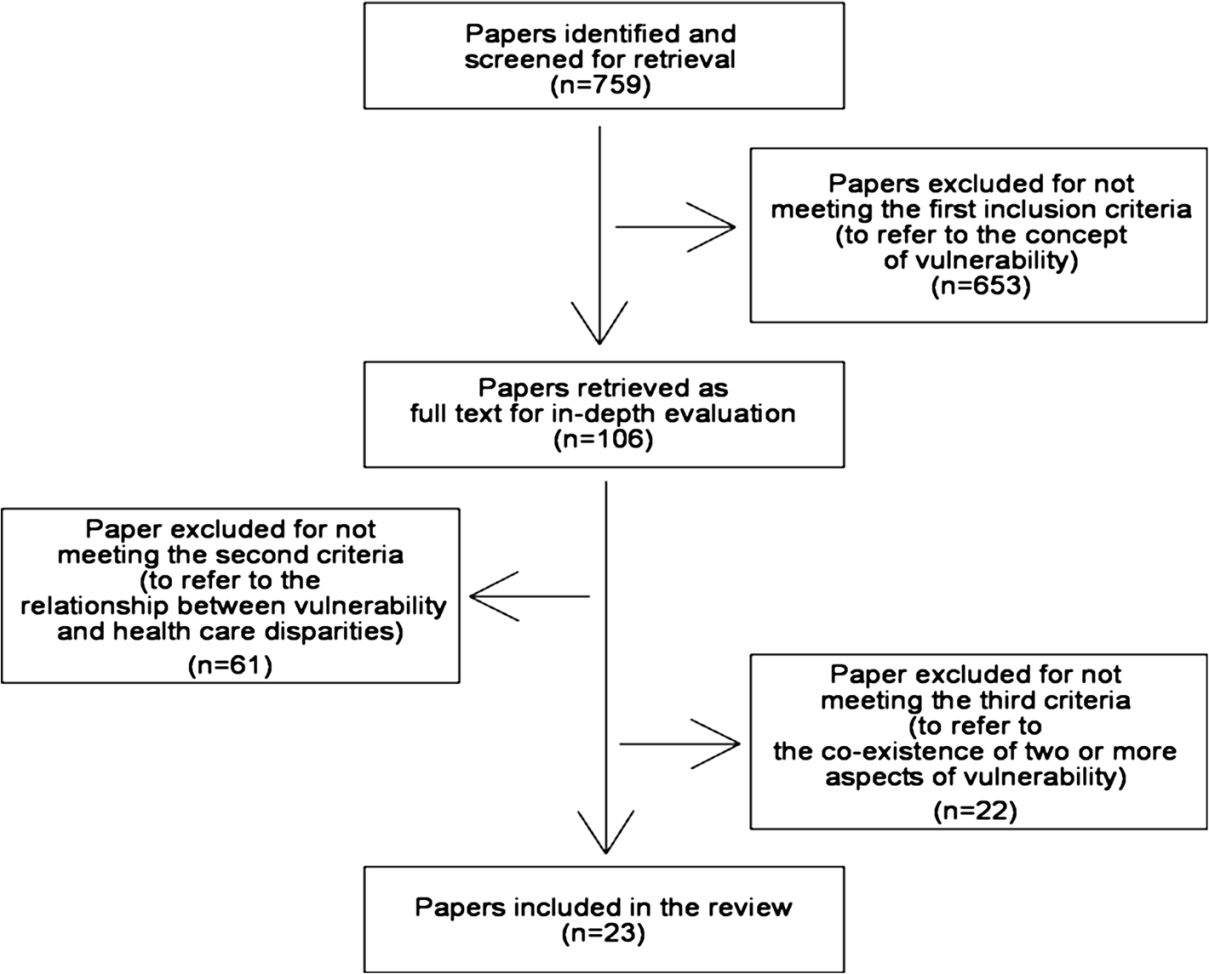
Flow diagram of search strategy and study selection process.

The search strategy generated 759 candidate references, of which 653 were excluded after the evaluation of their abstracts for not meeting the first inclusion criteria (not explicitly referring to the concept of vulnerability). Articles were selected for analysis only if their main focus was on vulnerability in reference to the personal and/or social condition of an individual or a group. Altogether, 106 articles were retrieved and read in full by the first author. After the in-depth evaluation, which aimed to assess whether or not all the inclusion criteria were satisfied, only 23 of these 106 papers were eventually included in the scoping review (Figure 2).

### Charting the data

The details of the publications included in the review are presented in Table 1. Each publication was first categorized based on: the type of approach (research report, which could be quantitative or qualitative, or discussion paper); language (English or French); and location of the study. Second, from each paper we extracted data related to: the study population; the main objectives; the vulnerability factors taken into consideration by the study; and the main findings (Table 2).

**Table 1:** Details of the publications included in the review

**Paper characteristic**	**Included papers, n (%)**
*Type*	
Quantitative research report	13 (56.5)
Qualitative research report	-
Discussion paper	10 (43.5)
*Language*	
English	20 (87)
French	3 (13)
*Paper Origin*	
United States	18 (78.3)
Switzerland	4 (17.4)
Canada	1 (4.3)
*Publication year*	
1990-1994	-
1995-1999	3 (13)
2000-2004	10 (43.5)
2006-2012	10 (43.5)

**Table 2:** Data charting

**Citation (type, language, location)**	**Study population**	**Main objectives**	**Vulnerability factors involved**	**Main findings**
1. Bieler *et al.,* 2012 (RS, QN, EN, Switzerland)	Patients ofan Emergency Department (ED)	To identify the social and medical vulnerability factors associated with ED frequent use	An accumulation of different social and medical factors	ED frequent users are more likely to accumulate social and medical vulnerability factors
2. Broyles, McAuley & Baird-Holmes,1999 (RS, QN, EN, USA)	Poor and uninsured elders	To assess health status and use of physician care of the medically vulnerable	Old age associated with illness, poverty and lack of insurance	Vulnerable elders are more likely to experience unmet medical needs and less likely to see a physician
3. Broyles, Narine & Brandt, 2000 (RS, QN, EN, USA)	Elders, poor (Medicaid beneficiaries) and uninsured people reporting a poor or fair health status	To assess the use of hospital care by the medically vulnerable	Illness associated with old age, poverty and lack of insurance	Vulnerable elders who reported poor or fair health were less likely to experience hospitalization and consumed fewer days of service
4. Carlson & Blustein, 2003 (RS, QN, EN, USA)	Enrollees in commercial HMOs (Health Maintenance Organizations)	To assess access to care among vulnerable populations enrolled in commercial HMOs	Low income and education associated with ethnicity and poor health	More vulnerable enrollees were more likely to report greater difficulties in seeing a specialist, obtaining help by telephone and getting tests or treatment
5. Denberg et al., 2006 (RS, QN, EN, USA)	African Americans with low-income and/or widowed	To assess the influence of patient race and social vulnerability on urologist treatment recommendations in prostate carcinoma	Race associated with low- income and widow status	More vulnerable patients experienced lower rates of recommendation for aggressive therapy
6. German & Latkin, 2012 (RS, QN, EN, USA)	Low-income women (96 % of the study participants were primarily African-American) at risk for HIV	To evaluate the role of accumulated vulnerability in association with HIV-related risk behaviors	Homelessness, incarceration, low-income, as indicators of social (in)stability	Each vulnerability indicator was significantly correlated with at least one HIV risk
7. Giger et al., 2007 (DP, EN, USA)	Racial, ethnic, uninsured, underserved, and underrepresented populations residing throughout the United States.	To discuss the development of cultural competences to eliminate health disparities	Poverty, belonging to a racial/ethnic minority, old age	Health and health care disparities could be eliminated by the development of specific knowledge, skills and competencies among health care professionals.
8. Fiscella & Shin, 2005) (DP, EN, USA)	Low-income persons, racial and ethnic minorities, the insured, etc.	To review disparities in health status and access to healthcare for vulnerable populations.	Low SES, belonging to a racial/ethnic minority, lack of insurance chronic illness, residence in underserved areas.	Healthcare policies do not adequately confront the paradox of the inverse care law, therefore disparities persist and, in some instances, actually worsen.
9. Mechanic & Tanner, 2007 (DP, EN, USA)	The poor and people with low education, ethnic minorities, inmates, people with physical and cognitive impairments.	To discuss the influence of values on how the society views the vulnerable and implications on health assistance.	A combination of individual and community dimensions	Limited access to high quality medical care is due to inadequate healthcare policies.
10. Monod & Sautebin, 2009 (DP, FR, Switzerland)	Older adults	To discuss elders’ vulnerability factors	Old age associated with loss of autonomy, multimorbidity, social exclusion and poverty	Older adults are suffering from limited access to care
11. Pauly & Pagán, 2007 (RS, QN, EN, USA)	People who are less likely than average to obtain medical care of an appropriate quality and quantity - the uninsured	To determine how the uninsurance rate is positively associated with lower quality care for the insured (negative spillover)	Poverty, ethnic minority, lack of insurance, chronic health conditions, psychiatric disorders	There are negative spillover effects from the uninsured to the insured in terms of the quality of health care, as a result of the low demand for quality by the uninsured
12. Pitkin Derose, Escarce & Lurie, 2007 (DP, EN, USA)	Immigrants in the United States	To discuss the sources of vulnerability to inadequate health care in immigrants	A combination of factors involving socio-political marginalization and a lack of socioeconomic and societal resources	Immigrants have reduced access to both personal medical services and public health services and programs (e.g. immunizations)
13. Rieder et al., 2010 (DP, FR, Switzerland)	Inmates	To discuss sources of shared vulnerability between inmates and health professionals	Detainee status associated with illegal immigration and psychiatric troubles	There are difficulties in access to health care in prisons in conditions of overcrowding and related to the lack of flexibility of prison functioning
14. Rogers, 1997 (DP, EN, Canada)	The poor, homeless, chronically ill and disabled, frail elderly people, immigrants and refugees.	To consolidate the available material on vulnerability and to introduce a vulnerability model for nurses’ use.	A combination of personal and environmental components.	The vulnerable experience reduced access to essential health care due to financial or social barriers.
15. Ruiz & Egli, 2010 (DP, FR, Switzerland)	Patients with diabetes and other chronic diseases	To discuss the metabolic syndrome in relationship with socio-cultural determinants	Chronic conditions related to socio-cultural factors such as poverty and ethnicity	Healthcare policies should take into consideration the sociocultural characteristic of patients
16. Shi, Forrest, von Schrader & Ng, 2003 (RS, QN, EN, USA)	Civilian, non-institutionalized persons in the 48 contiguous states of the United States	To examine whether patients’ perceptions of their relationships with primary care practitioners vary by vulnerability status	A combination of predisposing, enabling and need attributes of risk	Racial disparities were identified in office waiting time and having a specific clinician at the primary care site.
17. Shi & Stevens, 2005a (RS, QN, EN, USA)	White adults and adults belonging to racial and ethnic minorities.	To present a profile of risk factors for poor access based on income, insurance coverage, and having a regular source of care	A combination of predisposing and enabling characteristics.	Individuals with combinations of risk factors are more likely to delay medical care.
18. Shi & Stevens, 2005b (RS, QN, EN, USA)	Individuals 18 years and older who completed a survey	To operationalize vulnerability as risk profiles of pre-disposing and enabling factors, and to determine their association with preventive care	A combination of predisposing and enabling characteristics	Each additional vulnerability risk factor was associated with a lower likelihood of receiving preventive services
19. Shi & Stevens, 2007 (RS, QN, EN, USA)	The uninsured and Medicaid insured	To examine the primary care experiences of uninsured and Medicaid patients	Poverty, ethnicity, lack of insurance, chronic illness	Vulnerable people experience greater disparities in primary care (in terms of access, continuity and comprehensiveness)
20. Shi, Stevens, Faed & Tsai, 2008 (DP, EN, USA)	Those at greater risk for poor health status and without adequate potential access to care: ethnic minorities, low income and uninsured populations	To introduce and discuss a general model of vulnerability	A combination of community-level and individual risk factors	Vulnerable populations experience limited regular access to health care and preventive services.
21. Stone, 2002 (DP, EN, US)	African Americans who have Medicare or other healthcare coverage	To summarize recently published data about healthcare disparities experienced by African Americans	A combination of race and poverty	Vulnerable populations should be proportionally represented at all levels of decisions that affect health care and that are aiming to eliminate healthcare disparities
22. Stevens, Seid, Mistry & Halfon, 2006 (RS, QN, EN, USA)	Children and adolescents 0–19 years old.	To analyze vulnerability as a profile of multiple risk factors for poor pediatric care based on race/ethnicity, poverty status, parent education, and insurance status	Childhood associated with poverty, belonging to a racial/ethnic minority, being uninsured, having parents with a low level of education	Higher risk profiles were associated with greater barriers to accessing primary care for children in ‘fair or poor’ health. Vulnerable children who have the greatest health care needs also have the greatest difficulty obtaining primary care.
23. Walker et al., 2010 (RS, QN, EN, USA)	Middle-aged and older adults living in a multiethnic, low-income area	To assess the disparities in health care related to age, low-income and belonging to a racial/ethnic minority	A combination of predisposing, enabling and need factors	Middle-aged and older adults who are uninsured and in poor health reported more problems receiving needed medical care or preventive services.

Legend : RS: Research study; QN: Quantitative Research Report; DP: Discussion Paper;

EN: English, FR: French.

### Summarizing and reporting the results

A narrative synthesis approach allowed us to elicit the common themes that emerged from the findings. The themes concerned the use of the concept of vulnerability, the health care disparities taken into consideration, and the relationship between the co-existence of multiple aspects of vulnerability and health care disparities.

## Results

### Literature profile

Of the 23 publications included in the scoping review, 13 (56.5%) were categorized as quantitative research studies and 10 (43.5%) were discussion papers (Table 1). No qualitative research satisfied the criteria. Most papers were written in English (87%), and the publications emanated largely from the United States (78.3%), with additional contributions from Switzerland (17.4%) and Canada (4.3%). Over the past 12 years, there was a considerable increase in studies meeting the inclusion criteria (87% published between 2000 and 2012 vs. only 13% published between 1990 and 1999).

### The use of the concept of vulnerability

About one half of the reviewed papers (n = 12) defined vulnerability by reference to the segments of the population considered as being vulnerable, without explicitly taking into consideration the co-existence of different risk factors [25-36]. The other half of the publications (n = 11) included papers that explicitly defined vulnerability as an accumulation of several interrelated dimensions (multi-vulnerability) [10,21,22,37-44]. Most of these papers were theoretically grounded on the behavioral model of health services use [45,46]. In this model, the use of medical care is posited to be dependent on several “predisposing”, “enabling”, and “need” variables, and vulnerability is operationalized as a convergence of those variables. In the reviewed papers, the predisposing factors referred to socio-demographic characteristics such as old age [40-42], belonging to an ethnic minority [10,21,41-44], low education level [44], and living in disadvantaged communities [10].

The enabling components considered were lack of insurance [10,21,40-42,44], poverty status [10,40-44], and lack of a regular source of care [10,21,44]. The need component was measured by the number of days of disability, self-reported history of illness [40-42], and self-rated health status [43].

### Health care disparities

**Figure 3:**
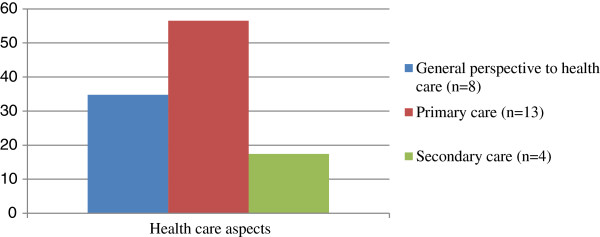
Aspects of the health care disparities considered by the reviewed papers.

The reviewed publications either approached disparities related to care from a general perspective, without focusing on specific aspects of health care, or addressed the disparities associated with primary or secondary care (Figure 3).

A majority of the included papers addressed primary care by referring to at least one of the attributes of primary care defined by the Institute of Medicine (i.e. accessibility, comprehensiveness, coordination, continuity, and accountability) [47,48]. Most publications referred to the attributes of accessibility, continuity, and comprehensiveness, with no article addressing coordination and accountability.

In regard to their approach to accessibility in relation to health care disparities, in some of the papers access to care was only mentioned, without any definition or explanation, as part of a broader discussion [28,33,35,36,38]. However, other publications took into consideration specific aspects of accessibility in connection with the context of vulnerability. Thus, access to health care was measured by the use or nonuse of physician care and, among users, by the number of visits [27,30,32,39,40]. Continuity of health care was measured as self-reports of having a regular source of care [27,30,32,39,43,46]. In turn, comprehensiveness was usually viewed in connection with difficulties with access to preventive services such as immunizations, check-ups, and screenings [27,30,35,39,42,44].

The papers concerned with secondary care focused on the difficulties in seeing a specialist when patients thought they needed one [32,42], on differences regarding the complexity and aggressiveness of cancer treatment depending on the patient’s socioeconomic status [25], and on uneven distributions of hospital care for vulnerable populations [41].

Finally, the publications with a general approach to health care discussed the differences in general quality of health care experienced by vulnerable populations [10,21,26], usually as a result of financial or social barriers [21,22,34,37]. They did not focus on aspects of primary nor secondary care, and the variable mentioned was general access to treatment [10,21,22,26,29,31].

### Multi-vulnerability and health care disparities

All the reviewed papers referred to the co-existence of different aspects of vulnerability that were linked, in one way or another, to diverse forms of health care disparities. Poverty status was considered as a major source of vulnerability by almost all papers (Table 3), and most of the papers referred to statistics that linked poverty with belonging to a racial/ethnic minority [10,21,22,25-27,29-32,34-36,38,39],[42-44].

**Table 3:** Aspects of vulnerability considered by the reviewed papers

**The aspects of vulnerability**	**Included papers, n (%)**
**Poverty**	21 (91.3)
**Racial/Ethnic minority**	18 (78,3)
**Chronic physical or mental illness**	12 (52.2)
**Lack of insurance**	8 (34.8)
**Old age**	6 (26)
**Incarceration**	3 (13)
**Immigrant status**	3 (13)
**Low level of education**	3 (13)
**Residence in underserved areas**	2 (8.7)
**Unemployment**	1 (4.3)
**Widowed status**	1 (4.3)
**Homelessness**	1 (4.3)

After poverty and racial/ethnic minority, three other aspects were most frequently taken into consideration in the conceptualization of vulnerability: old age [22,26,33,40-42], lack of insurance [10,26,27,30,34,37,40,41], and the presence of chronic physical or mental illnesses [22,27-30,33,34,37,38,40-42].

Finally, other factors associated with vulnerability were incarceration [28,36,38], homelessness, and residence in underserved areas [10,27], as well as some aspects related to socioeconomic status: migration [22,28,35], low level of education [32,38,39], unemployment [37], and widow status [25].

All findings mentioned above point to the fact that people who accumulate more vulnerability factors are more likely to face health care disparities. Thus, the papers conclude that individuals who present the most vulnerability aspects are the most likely to: report difficulties in seeing a specialist [30,32]; get less aggressive treatment for cancer [25]; and face greater barriers to access to quality primary care [26,27,33,34,36-39].

Almost one-third of these papers operationalized vulnerability as a convergence of factors, as described by Andersen and Aday [46] in the behavioral model of health services use. These studies’ findings suggest that a combination of predisposing, enabling and need factors is more likely to result in a distribution of health care that is incongruent with the medical needs of vulnerable populations [40-42]. Moreover, the groups that accumulate the most vulnerability aspects are more likely to face care disparities such as longer office waiting time [43] and delayed needed medical care because of affordability [10,21], and are less likely to have a regular source of care [43] and to get preventive services [10,44].

## Discussion

The goal of this paper was to present the results of a scoping review on the concept of vulnerability in connection with health care disparities. Moreover, we intended to identify what is known, in the existing literature, about the relationship between health care disparities and the co-existence of multiple aspects of vulnerability. The main finding of this scoping review is that the body of literature on vulnerability in health care research confirms the framework of our dynamic vulnerability model of health care disparities based on the ICL. Thus, the results suggest that high levels of vulnerability (due to the co-existence of multiple vulnerability aspects) would increase health care needs and would be associated to lower health care accessibility and quality. These findings are consistent with other research showing that the ICL phenomenon holds even under universal health insurance systems such as the UK and Canada, and it holds with greater force in countries without universal health insurance [18,19,27,49]. However, the total number of studies that operationalized vulnerability as a combination of factors is too small for drawing statistically firm conclusions regarding the relationship between the increase of co-existing vulnerability factors and the escalation of health care disparities. Hence, there is a need for more research into the scientific validation of this correlation. Moreover, we recognize the need for studies to identify the clusters of vulnerability aspects that increase the probability of facing health care disparities. This research will provide the policy-making process with currently missing information on how the ICL actually operates and will eventually help reduce inequalities in health and health care.

Our review revealed that despite its substantial additions, the existing literature has both important theoretical and empirical limitations. For instance, the vast majority of papers is mostly heuristic and lacks a solid theoretical basis. Even when based on a conceptual framework, the one that prevails is the initial behavioral model of health services use [46] that was often criticized and repeatedly revised to emphasize the dynamic and recursive nature of health care use [45,50]. The reviewed research studies also have some important methodological limitations related mostly to their reliance on survey data, which could preclude causal interpretation and only measure statistical associations and tendencies.

Our review also revealed that there is currently little research concerned with the health care disparities related to the co-existence of multiple aspects of vulnerability. Of the 759 references generated by the initial search, only 23 referred, explicitly or implicitly, both to health care disparities and to the co-existence of two or more aspects of vulnerability. Moreover, less than half of these reviewed publications provided empirical evidence on the relationship between health care disparities and the co-existence of multiple vulnerability factors. When taken into consideration, this connection referred mostly to demographics and social structure, ignoring health beliefs and patients’ experience of illness. According to several authors, health and illness beliefs are of great importance in explaining the use of medical and preventive services, as they directly affect need and, consequently, services use [45,51,52].

The fact that none of the included papers addressed the beliefs or conceptions about health and illness of vulnerable populations is, in our opinion, the major gap in the reviewed literature. In fact, all the reviewed publications approached vulnerability from a normative perspective based on socio-demographic characteristics that assign certain populations a higher probability of health or health care disparities. Spiers [53] contrasts this kind of approach, which she names “etic”, with an “emic” perspective on vulnerability. Inspired by the anthropological literature, the emic approach to vulnerability refers to a description of the phenomena as perceived by the concerned person and reflects the lived experience of “vulnerable” populations. According to the author, research on vulnerability would greatly benefit from the integration of the emic dimension to the etic approaches to vulnerability, as this combined perspective would provide a more satisfactory picture of how people manage multiple challenges in their daily lives [53].

The present review also revealed a series of additional gaps in the literature that could be interpreted as opportunities for future research. These gaps pertain to location and type of studies, and the aspects of vulnerability related to health care disparities. Regarding location, the vast majority of the publications were conducted in the United States, where there is no universal health care system to sustain appropriate care for vulnerable populations. However, there is evidence that health care disparities on the basis of poverty, race and immigrant status persist even in countries with a universal health care system [54]. Therefore, we argue that future research conducted in countries with a publicly funded health care system (e.g., Canada, the UK) would increase our understanding and, eventually, contribute to the reduction of these disparities.

In regard to the types of studies, no qualitative research satisfied the inclusion criteria of our scoping review. Besides the discussion papers that addressed vulnerability and health care disparities from a theoretical perspective, the research papers exclusively reported findings from quantitative studies. Even though these studies might present the usual strengths of quantitative research (e.g., structured research design, generalization of findings, statistical significance, and objectivity of the researcher), they also have weaknesses. Without entering into a debate over the advantages and disadvantages of both types of research, we argue that studies on health care disparities related to vulnerability could only benefit from the integration of more flexible qualitative techniques in their design.

Concerning the aspects of vulnerability involved in health care disparities - with respect to the community social resources model [24] - our review has shown that the vast majority of papers focused on the lack of socioeconomic resources; namely, human capital (i.e., poverty, low level of education, unemployment, and homelessness) and social connectedness (i.e., racial/ethnic minority, old age, immigrant status, and widowed status). Only a few studies took into consideration the aspects related to environmental resources (i.e., residence in underserved areas) and none of them referred to the vulnerability to hazards and disaster events (see Table 3). Since the likelihood of a hazard event combined with the lack of socioeconomic resources results in higher degrees of vulnerability and increased needs of people living in certain communities [55-57], we recognize the need for research that examines the link between this “vulnerability of places” [55] and health care disparities.

Our review also revealed a paucity of studies envisaging the close connection between chronic illness and health care disparities, even though it has already been suggested that people living with chronic conditions may experience challenges when navigating through the health care system [58-60]. Moreover, the co-existence of two or more chronic diseases in the same person (i.e., multimorbidity) as a vulnerability aspect was almost absent from the publications reviewed.

The prevalence of multiple chronic conditions in the same individual has dramatically increased over the past years and is starting to be recognized as a critical clinical issue in health care, mostly because of its negative effects on patient quality of life, mortality, and treatment complications [61-63]. Several studies also found that individuals with multimorbidity have trouble obtaining quality care, usually in terms of access and coordination [64,65]. However, research on multimorbidity is still in its infancy [66] and our scoping review revealed that there are very few studies considering multimorbidity as a vulnerability factor in connection with health care disparities. Further research addressing health care disparities related to multimorbidity that co-exists with other vulnerability factors (e.g., poverty, belonging to an ethnic minority, etc.) might lead to crucial information on how to transform typical primary care practices to meet the needs of the most vulnerable patients.

Another vulnerability aspect that has received little attention in the research literature is immigrant status. Even though a large majority of the papers considered racial/ethnic minority as a vulnerability factor, health care disparities experienced by immigrants involve more complex interrelated issues because of other vulnerability aspects than ethnicity. Thus, several studies found that even within the same racial/ethnic group, immigrants received significantly less medical and preventive care than their non-immigrant counterparts [67-69]. Among the potential reasons that account for these findings are: language and cultural barriers [70,71], disparities in health care insurance [72], and a lack of familiarity with the local health care system [73].

The above-mentioned problems are frequently combined with chronic poverty, especially for recent immigrants [74,75], and with an increase in the prevalence of chronic diseases [76,77]. All these aspects lead naturally to the conclusion that immigrant status is an important vulnerability aspect that often co-exists and may synergistically interact with other recognized factors involved in health care disparities. We consider that further research addressing these interactions would be beneficial for finding solutions aimed to overcome such disparities.

Before concluding, it is important to raise some limitations of our scoping review. First, we restricted the search strategy to databases that usually cover the health, public health and psychological literature (i.e. MEDLINE-Ovid, CINAHL, EMBASE and PsycINFO). For practical reasons, we were unable to exhaustively search the social sciences databases and to include papers in a language other than English or French. Therefore the risk that not all relevant studies were identified remains. Second, we located and analyzed only 23 publications on the topic, which precludes a comprehensive assessment of such a complex domain. These 23 papers tended to be rather diverse in both their focus and design, which led us to make decisions regarding the information to be summarized and analyzed. Furthermore, since scoping reviews do not typically include a quality assessment of included studies, data synthesis and interpretation could be limited [78,79] and the evidence to base decisions regarding the need for future research could be insufficient [15]. However, we are confident that our scoping review provided original data with a richness and fineness that would have been difficult to attain with other types of literature reviews.

## Conclusions

This scoping review confirmed our hypothesis of the direct correlation between the increase of co-existing vulnerability factors and the escalation of health care disparities. However, it also revealed that there is currently little research concerning health care disparities related to the co-existence of multiple aspects of vulnerability. Several gaps in the literature were identified, among which the most important seem to be a lack of research focusing on vulnerable populations’ perception of their own vulnerability, and on multimorbidity and immigrant status as aspects of vulnerability.

We argue that future research addressing these gaps would help foster primary care interventions that are responsive to the needs of vulnerable people and, eventually, contribute to the reduction of health care disparities in society.

## Abbreviations

ICL: Inverse Care Law

## Competing interests

The authors have no financial or non-financial competing interests.

## Authors’ contributions

CG conceived the study, realized the selection of papers, participated in the design of the study and drafted the manuscript, tables and figures. CL coordinated the study, participated in its design and helped to draft the manuscript. MF helped with the study coordination and drafting the manuscript. All authors read and approved the final manuscript.

## Pre-publication history

The pre-publication history for this paper can be accessed here:

http://www.biomedcentral.com/1472-6963/13/94/prepub
